# Spatial heterogeneity in glioblastoma: linking single-cell and spatial multi-omics to vaccine immunotherapy

**DOI:** 10.3389/fimmu.2026.1921816

**Published:** 2026-10-12

**Authors:** Juan Yuan, Yong Liu

**Affiliations:** 1 Department of Cell and Molecular Biology, Karolinska Institutet, Stockholm, Sweden; 2 Department of Laboratory Medicine, Karolinska Institutet, Stockholm, Sweden

**Keywords:** antigen heterogeneity, cancer vaccines, glioblastoma, spatial multiomics, tumor microenvironment

## Abstract

Glioblastoma (GBM) remains the most lethal primary brain tumor, with survival still limited despite advances in multimodal treatment. The tumor’s profound heterogeneity--across patients, within individual tumors, and over time--has frustrated every class of therapeutic intervention. Recent developments in single-cell and spatial omics technologies have substantially advanced our understanding of this heterogeneity. No longer viewed as a uniform mass, GBM is now recognized as a spatially organized ecosystem of distinct malignant cell states, immune neighborhoods, and metabolic niches that interact across macroscopic distances. Spatial transcriptomics has revealed that malignant cells exist along a continuum of transcriptional programs--from neural progenitor-like to mesenchymal-like--that are not randomly distributed but organized into structured regions with a multi-layered architecture associated with hypoxia. Spatial proteomics and imaging mass cytometry have complemented these findings by mapping protein-level immune landscapes, uncovering cellular neighborhoods associated with survival and identifying spatially defined immunosuppressive niches. This review summarizes current knowledge from single-cell and spatial multi-omic studies of GBM, with an emphasis on the spatial organization of malignant cell states, the architecture of the tumor immune microenvironment, and the implications of these findings for therapeutic development, particularly vaccine immunotherapy. We argue that spatial technologies do not replace existing therapeutic pipelines but address a critical blind spot: the spatial dimension that bulk measurements cannot see.

## Introduction

Glioblastoma is still the most common malignant primary brain tumor in adults. In the landmark chemoradiotherapy trial, median overall survival was 14.6 months (1), and prognosis remains poor despite subsequent therapeutic advances. Profound cellular and molecular heterogeneity contributes substantially to therapeutic resistance and the near-universal recurrence of GBM (2).

The failure of therapeutic development in GBM is fundamentally a failure to contend with heterogeneity. Advances in single-cell transcriptomic and proteomic profiling, together with spatial imaging approaches, have revealed extensive heterogeneity in GBM, including both inter-patient diversity and intra-tumoral cellular variability. Early bulk transcriptomic profiling classified GBM into four expression subtypes--proneural, neural, classical, and mesenchymal (3). Subsequent tumor-intrinsic analyses indicated that the neural category largely reflected non-neoplastic neural-lineage contamination and refined the framework to three programs--proneural, classical, and mesenchymal (4). For consistency, unless otherwise specified, subsequent references in this review to bulk transcriptional subtypes denote these three tumor-intrinsic programs. Single-cell RNA sequencing further resolved GBM diversity by showing that oligodendrocyte progenitor-like (OPC-like), neural progenitor-like (NPC-like), astrocyte-like (AC-like), and mesenchymal-like (MES-like) malignant cell states coexist within individual tumors (5).

Single-cell RNA sequencing first exposed the extent of this heterogeneity at unprecedented resolution. It revealed that GBM is not a monolithic entity but a complex ecosystem of malignant and non-malignant cells that interact in intricate ways. Spatial omics technologies have now added a critical dimension to this understanding, enabling the molecular characterization of regions or individual cells within their spatial context and providing previously unattainable insights into the complex organization of the GBM tumor microenvironment. Histological heterogeneity in GBM has long been recognized, with different tumor regions showing distinct patterns of cellular organization. Hans-Joachim Scherer’s pioneering studies linked these spatial patterns to glioma progression and invasion (6).

Given the breadth and rapid development of GBM spatial biology, this mini review is not intended to provide an exhaustive survey of all spatial studies. Instead, it focuses on single-cell and spatial multi-omic findings most directly relevant to vaccine immunotherapy, including spatial antigen heterogeneity, malignant-cell-state plasticity, immune-cell accessibility, and regional immunosuppression. The review focuses on adult GBM; pediatric and adolescent gliomas may differ in their molecular drivers, developmental context, and therapeutic considerations, and therefore warrant a dedicated review beyond the present scope.

## The cellular states of GBM: from bulk subtypes to single-cell landscapes

For years, GBM was classified into transcriptional subtypes based on bulk tumor expression profiling. The original TCGA-based classification described proneural, neural, classical, and mesenchymal subtypes (3), whereas subsequent tumor-intrinsic analysis refined this framework to proneural, classical, and mesenchymal programs (4). Rather than discrete fixed subtypes, single-cell studies have replaced bulk TCGA “subtypes” with a dynamic model in which multiple malignant programs interconvert, and the balance among these programs is shaped by genetic lesions and local ecological cues (5).

Neftel and colleagues established the foundational framework, demonstrating that malignant cells in GBM adopt four recurrent transcriptional programs that recapitulate distinct neural cell types: OPC-like, NPC-like, AC-like, and MES-like (5). Crucially, these are not fixed identities but plastic states shaped by microenvironmental cues and genetic drivers. A malignant cell can transition between states in response to therapy, immune pressure, or local signals (5, 7, 8).

Richards and colleagues extended this framework by showing that GBM stem cells map along a transcriptional gradient spanning developmental and injury-response programs (9). This developmental-injury response gradient defines functional vulnerabilities that differ across the spectrum, suggesting that glioblastomas may grow out of a fundamental stem cell-based neural wound response program. The implication is direct: an intervention that targets one state may leave cells in another state untouched, providing a reservoir for recurrence.

Single-cell studies indicate that transcriptional heterogeneity in GBM reflects both cell-intrinsic and cell-extrinsic influences. However, single-cell RNA sequencing alone cannot determine whether tumor cell states are randomly distributed or preferentially associated with regional microenvironments. This unresolved question has motivated the application of spatial technologies to GBM.

## Spatial organization of malignant cell states

The most transformative insight from spatial transcriptomics is that cellular states are not randomly distributed but organized across space. Greenwald and colleagues combined spatial transcriptomics, spatial proteomics, and computational approaches to define glioma cellular states and uncover their organization (10). Their work revealed that human glioblastoma consists of both disorganized and structured regions. Structured regions display a five-layered organization that is associated with hypoxia and extends beyond the organization visible by histology (10).

This five-layered architecture represents a major advance in our understanding of GBM spatial biology. The organization of cell states is associated with the abundance of hypoxic cancer cells, and recurring pairs of states define a five-layer organization in structured regions. Hypoxia functions as an important tissue organizer, patterning cellular states across macroscopic distances (10). Tumor regions distant from hypoxic or necrotic foci--and tumors that lack marked hypoxia--show less evidence of this structured organization, supporting a central role for hypoxia in this form of spatial patterning (10).

The relationship between hypoxia and immune cell distribution is spatially complex. Greenwald and colleagues delineated hypoxia-associated layers within the structured GBM architecture (10). Hypoxic niches, however, should not be treated as simply T-cell-free compartments: in experimental model systems, spatial analyses have shown that tumor-associated macrophages and cytotoxic T lymphocytes can be recruited to and retained within these niches, where they are reprogrammed toward immunosuppressive states (11). Thus, hypoxia may restrict effective immunity through immune-cell retention and functional suppression rather than spatial exclusion alone.

Ravi and colleagues used spatially resolved transcriptomics, metabolomics, and proteomics to characterize GBM (12). By deciphering regionally shared transcriptional programs across patients, they inferred that GBM is organized by spatial segregation of lineage states and adapts to inflammatory or metabolic stimuli, reminiscent of the reactive transformation in mature astrocytes (12). Their work demonstrated that spatially segregated lineage states adapt regionally to inflammatory and metabolic stimuli--meaning that a therapeutic target expressed in one region may be absent from another.

A recently reported human GBM atlas combined spatial gene-expression profiling across tissue sections with matched single-cell and protein measurements at subcellular resolution (13). In its Xenium component, a targeted 348-gene panel enriched for vascular and stromal markers enabled the characterization of less well-described endothelial, perivascular, and fibroblast-like cell states and their relationships with malignant and immune compartments (13). The analysis also identified a distinct oligodendrocyte population localized to the tumor core and perivascular regions, with a gene-expression profile associated with recurrence and poor clinical outcome (13). Its public availability makes the atlas a useful resource for examining GBM tissue organization and prioritizing region-specific cellular interactions for therapeutic investigation.

Several recent atlas-like resources have further expanded the field without changing the central argument of this review. These include a spatial transcriptomic map of coordinated GBM tissue architecture (14), single-cell spatial profiling of the GBM matrisome (15), and an integrated invasion-resource study linking GBM cell states to peri-axonal and peri-vascular invasion programs (16). Together, these resources strengthen the conclusion that malignant states, stromal programs, and invasive niches should be interpreted in their anatomical context rather than only through bulk tumor averages.

Other studies have identified niche-specific enrichment and vulnerabilities of specific cell populations. Spatial transcriptomics has revealed that tissue stem cell signatures are significantly enriched in regions bordering necrosis and the peritumoral area, positively correlated with the mesenchymal subtype signature (14). This finding suggests that the most aggressive, stem-like cells are not uniformly distributed but concentrated in specific anatomical niches.

Recent work has also characterized the spatial organization of the extracellular matrix in GBM. Using single-cell spatial transcriptomics, De et al. profiled nearly 400 ECM-related genes in GBM and non-cancerous brain samples (15). The analysis distinguished at least four GBM cell populations with different ECM programs and preferential localization to separate intratumoral regions (15). ECM signatures showed limited spatial overlap across stromal populations, particularly vascular endothelial cells and microglia/macrophages. Computational inference further identified previously unrecognized ECM ligand–receptor relationships between stromal and GBM cells, offering insight into how the matrix may influence brain tumor initiation and progression (15).

Collectively, these studies establish that GBM is not a well-mixed suspension of cells but a spatially compartmentalized organ. Any therapy that depends on cell-cell interactions must contend with this spatial reality. For vaccine immunotherapy, the relevant question is therefore not simply whether an antigen is present in GBM, but whether it is distributed across malignant states and anatomical regions likely to be encountered by immune effector cells.

## Invasion programs and the spatial niche of invasive cells

Invasion of GBM cells into the surrounding brain remains incompletely understood and poses a major barrier to treatment. The diffuse distribution of GBM cells across the brain is a major obstacle to treatment. Gross total resection can extend recurrence-free survival, yet malignant cells beyond the clinically detectable tumor boundary remain and ultimately give rise to recurrence.

Recent work combined single-cell RNA sequencing, spatial transcriptomics, and multiplexed imaging in orthotopic xenograft models to examine GBM invasion (16). Screening 20 patient-derived gliomasphere models in mice showed enrichment of oligodendrocyte progenitor-like cells in models with distal invasion, whereas models showing only local invasion were enriched in mesenchymal-like cells (16). These phenotypes predominantly followed peri-axonal and peri-vascular routes, respectively, consistent with the Scherer secondary structures described in classical histopathology (6, 16, 17).

Transcriptomic comparison of invading cells with tumor-core cells distinguished programs linked to distal and local invasion from those expressed during the invasion process itself (16). Spatial transcriptomics and multiplexed imaging confirmed these findings and further localized the niches occupied by invasive cells (16). Together, these results connect GBM cell states and invasion-associated programs across spatial scales. Because these invasion-route findings were generated largely in orthotopic xenograft models, they should be interpreted as a mechanistic framework rather than direct clinical evidence for vaccine resistance.

GBM cells invade the brain parenchyma over both local and distant ranges, with interhemispheric white matter tracts providing a route to the contralateral hemisphere (6, 16, 17). Classical histopathological and molecular studies showed that malignant cells preferentially spread along existing anatomical structures, particularly white matter tracts (peri-axonal) and blood vessels (peri-vascular) (6, 17).The spatial technologies now available enable these classical histological observations to be placed on a molecular footing, revealing the transcriptional programs that underpin different invasion routes.

## Spatial proteomics: adding the protein layer

While spatial transcriptomics maps where genes are expressed, spatial proteomics reveals where proteins are present--a critical distinction for therapeutic applications. Greenwald and colleagues integrated spatial transcriptomics with spatial proteomics, demonstrating that protein-level validation can help assess the functional relevance of transcriptomic findings (10).

Imaging mass cytometry enables highly multiplexed, spatially resolved measurement of protein markers at single-cell resolution. In GBM, this approach has been used to quantify structural and cellular features of the tumor microenvironment and to identify clinically relevant cellular neighborhoods (18).

Karimi and colleagues applied imaging mass cytometry to characterize the immunological landscape of 139 high-grade glioma and 46 brain metastasis tumors (18). Their analysis revealed cellular neighborhoods associated with survival in patients with glioblastoma, identifying a unique population of myeloperoxidase (MPO)-positive macrophages associated with long-term survival (18). This finding demonstrates that spatial proteomics can uncover clinically relevant immune populations that may not be resolved by transcriptomic approaches alone.

Longitudinal spatial proteomic analysis has begun to clarify treatment-associated remodeling of the GBM microenvironment. In paired primary and recurrent tumors from five patients, imaging mass cytometry quantified 34 protein markers to resolve GBM-associated cell populations and their interactions at single-cell resolution (19). Following treatment, normal brain cells became more abundant whereas vascular cells decreased, accompanied by increased interactions involving astrocytes, oligodendrocytes, and vascular cells (19). Moreover, the hypoxia-linked layered pattern evident in primary tumors became less prominent at recurrence, where a less ordered architecture associated with reactive astrocytes and lymphocytes emerged (19).

A peer-reviewed mass spectrometry-based spatial proteomic atlas mapped 4,794 proteins across hallmark histomorphological niches in IDH-wildtype GBM, including leading edge, infiltrating tumor, cellular tumor, palisading cells around necrosis, and microvascular proliferation (20). The study identified regionally enriched proteins and molecular programs that were obscured in bulk-tissue profiles, illustrating why protein-level target nomination requires anatomically resolved sampling (20).

The integration of spatial transcriptomics and proteomics provides a more complete picture of GBM biology. Transcriptomics identifies which genes are potentially active; proteomics helps determine whether those genes are translated into proteins in the relevant spatial contexts. For vaccine antigens, however, direct evidence of HLA-bound peptide presentation still requires immunopeptidomic or antigen-specific validation.

## Spatial architecture of the immune microenvironment

The spatial organization of immune cells within GBM is as important as the organization of malignant cells. Karimi’s imaging mass cytometry study revealed that the GBM immune microenvironment consists of distinct spatial neighborhoods with different functional properties (18). These neighborhoods are not randomly distributed but organized in patterns that correlate with patient outcomes (18).

Coy and colleagues used single-cell spatial analysis to map immunomodulatory purinergic signaling in GBM (21). They found that GBMs enriched for CD73--the ectoenzyme that generates immunosuppressive adenosine--exhibit inflammatory microenvironments, suggesting that purinergic signaling regulates immune adaptation (21). Importantly, they showed that tumor CD73 and microglial CD39 exhibit strong spatial correlation, and this proximity is associated with poor outcome (21). The implication is direct: even if immune cells reach the tumor, they may encounter an adenosine-rich niche that suppresses their function.

Hara and colleagues demonstrated that macrophages and microglia induce the transition of GBM cells into a mesenchymal-like cellular state through oncostatin M signaling (7). This finding reveals that immune cells do not merely occupy space but actively remodel the malignant state composition in their vicinity. A mesenchymal-like state may be more therapy-resistant and may express a different set of antigens than other states. Immune pressure from a therapeutic intervention may therefore not simply kill tumor cells but actively reshape the tumor’s cellular composition--potentially selecting for more aggressive or therapy-resistant states. Wang and colleagues further showed that phenotypic plasticity is a major component of therapy-associated GBM evolution, suggesting that treatment pressure can drive state transitions that alter antigen expression (8).

Combined spatial transcriptomic and immunofluorescence analyses localized S100A9-high monocytes near M2 macrophages, exhausted CD8+ T cells, and endothelial cells (22). Such an immunosuppressive environment may contribute to GBM progression and resistance to treatment.This spatial adjacency of immunosuppressive cell types suggests that the physical architecture of the tumor may help maintain immune suppression.

In recurrent GBM, ECM reflectance imaging revealed an organized perivascular niche containing abundant immunosuppressive macrophages (23). Single-cell spatial transcriptomics further resolved macrophage populations specific to this niche and mapped their relationships with endothelial cells and perivascular fibroblasts (23). Together, these findings link recurrent-tumor evolution to genetic variation and spatially organized features of the tumor microenvironment.

Spatial transcriptomic analysis has also revealed a lack of measurable response to PD-1 blockade in recurrent glioblastoma (24). In the randomized phase III CheckMate 143 trial, nivolumab monotherapy did not improve overall survival compared with bevacizumab in recurrent GBM (25). A small randomized study in recurrent GBM compared neoadjuvant plus adjuvant pembrolizumab with adjuvant pembrolizumab alone; although it reported immune activation and a survival difference, both groups received PD-1 blockade and the trial was not designed to establish an overall-survival benefit (26). A recent spatial transcriptomic study reported that neoadjuvant PD-1 blockade did not elicit measurable responses at the spatial transcriptomic level in tumor cells or tumor-associated macrophages in recurrent GBM (24), suggesting that spatial organization of the tumor microenvironment may contribute to resistance.

Glioma-neuronal circuit remodeling has been identified as a driver of regional immunosuppression (27). Glioblastoma cell-derived TSP1 has been identified as a causal mediator of the suppressive immune microenvironment in the context of distinct patterns of glioma-neuronal circuit remodeling and neuronal activity (27). This finding adds another layer of complexity to the spatial immune landscape of GBM: not only are immune cells spatially organized, but neuronal activity itself shapes immune suppression in a region-specific manner. Together, these studies suggest that immune accessibility is a spatial property, shaped by perivascular organization, myeloid-rich niches, metabolic suppression, and neuronal activity, rather than by the total abundance of immune cells alone.

## Implications for vaccine immunotherapy: a spatial framework

The spatial organization of GBM--both of malignant cell states and of immune cells--has profound implications for vaccine immunotherapy. Two phase I trials published in Nature in 2019 established the feasibility of this approach. In the Keskin study, eight patients with newly diagnosed, MGMT-unmethylated GBM received personalized neoantigen vaccination after surgery and radiotherapy without temozolomide. Circulating polyfunctional neoantigen-specific CD4+ and CD8+ T cell responses were observed primarily in patients who did not receive dexamethasone during vaccine priming, whereas systemic responses were minimal in patients receiving the corticosteroid (28). Evidence linking peripheral vaccine-reactive clonotypes to intracranial tumors was obtained in only a small subset and should be interpreted accordingly (28). Hilf and colleagues, in the GAPVAC trial, showed that actively personalized vaccination combining unmutated tumor-associated antigens with predicted neoepitopes was feasible and immunogenic in newly diagnosed GBM (29).

Yet the phase III ACT IV trial of rindopepimut, a peptide vaccine targeting the GBM-specific EGFRvIII mutation, failed to improve overall survival despite compelling preclinical and early-phase immunogenicity data (30). EGFRvIII is tumor-specific and immunogenic, but single-nucleus sequencing has demonstrated substantial intratumoral heterogeneity of EGFR variants in GBM (31). It is therefore possible that rindopepimut-induced T cells encountered EGFRvIII-positive cells only in immune-excluded or suppressed niches, or that EGFRvIII-negative regions served as reservoirs for recurrence.

More recently, the DCVax-L phase III trial reported survival associations with autologous tumor lysate-loaded dendritic cell vaccination. The trial originally randomized patients 2:1 to DCVax-L or placebo and allowed crossover after progression; because most placebo-assigned patients subsequently received DCVax-L, the published overall-survival analysis used external control populations rather than the original randomized comparison. The original progression-free-survival endpoint did not demonstrate benefit, and the crossover plus externally controlled survival analysis limits causal inference regarding efficacy (32, 33). Latzer and colleagues reported a real-world observation of 173 patients with GBM treated with personalized peptide vaccines, demonstrating that approximately 90% of patients with immune monitoring mounted detectable immune responses, and multiple vaccine-induced T cell responses were associated with longer survival (34). However, this study was subsequently criticized for selection bias, lack of an intent-to-treat report, absence of a trial protocol, and lack of a controlled design (35).

Spatial heterogeneity is unlikely to affect all vaccine modalities in the same way. For private neoantigen vaccines, the main concern is whether selected mutations are clonal, expressed, and regionally conserved. For shared-driver vaccines such as IDH1(R132H)- or H3K27M-targeted approaches, spatial analysis may be more useful for assessing antigen presentation and immune accessibility than for identifying the antigen itself. For tumor-associated-antigen vaccines, spatial profiling can help distinguish malignant-cell expression from expression in normal or reactive cell types and evaluate state-spanning coverage. For tumor-lysate dendritic-cell vaccines, spatial omics cannot define a single antigenic target but may help interpret which tumor regions and immune niches contribute to response or escape.

What explains this gap between immunogenicity and clinical efficacy? We propose that a critical missing dimension is space. Current personalized neoantigen-selection pipelines rely predominantly on bulk genomic and transcriptomic profiling of resected tumor tissue. Candidate selection additionally incorporates HLA typing, peptide-MHC binding prediction, clonality, variant allele frequency, RNA abundance, and, in approaches such as GAPVAC, HLA ligandome analysis (29). These strategies are scientifically rigorous and clinically feasible, but they generally do not preserve the spatial coordinates needed to determine whether candidate antigens are conserved across malignant cell states and anatomical regions.

Spatial omics provides a way to interrogate several steps of the vaccine design pipeline in a spatially resolved manner. Spatial transcriptomics can assess whether a candidate antigen is expressed across tumor regions and malignant cell states. Spatial proteomics can test whether the encoded protein is present in relevant spatial contexts, while immunopeptidomics or antigen-specific assays are needed to confirm HLA presentation. Spatial immune profiling can determine whether T cells can access antigen-positive cells and whether the local microenvironment will support or suppress their function.

We propose that future studies test whether vaccine antigen selection can be improved by incorporating three spatial criteria: (1) state-spanning expression--does the antigen cover OPC-like, NPC-like, AC-like, and MES-like states, or is it restricted to a single state that could be lost through state switching? (2) regional conservation--is the antigen expressed in the tumor core, the invasive margin, and perivascular niches where T cells typically extravasate? (3) recurrent stability--is the antigen retained in paired recurrent samples after standard therapy, or does it disappear through plasticity rather than mutation?

The IDH1 vaccine developed by Platten and colleagues provides an instructive contrast (36). IDH1(R132H) is a shared clonal neoantigen: tumor-specific, a driver mutation, clonal, and absent from normal tissue. The vaccine induced mutation-specific T helper cell responses in newly diagnosed glioma patients (36). This is the kind of “ideal” antigen that spatial omics might help identify in IDH-wildtype GBM--if such antigens exist. Similarly, the H3K27M-targeted vaccine for diffuse midline glioma provides an example of immunogenic vaccination against a shared driver mutation (37); that study did not spatially map antigen distribution.

For IDH-wildtype GBM, which lacks such cleanly defined shared neoantigens, spatial approaches become even more critical. The field must move from antigen discovery to spatial antigen ecology--an approach that evaluates vaccine targets not by mutation or expression alone, but by their distribution across space, their stability across cell states, and the accessibility of the niches in which they reside.

## Technical considerations and future directions

Several technical considerations merit discussion. First, spatial transcriptomics measures RNA, not protein or peptide presentation. An antigen may be transcribed in a region but not translated, processed, or presented on HLA. Spatial proteomics and immunopeptidomics are required to bridge this gap, and these technologies are currently less mature and less spatially resolved than transcriptomic methods.

These modalities provide different levels of evidence: spatial transcriptomics nominates RNA-level candidates and regional programs; spatial proteomics or multiplex imaging validates protein abundance and cellular context; immunopeptidomics provides direct evidence of HLA-bound peptides; and TCR-based assays are needed to link antigen recognition to functional T-cell responses.

Second, the clinical feasibility of pre-vaccination spatial profiling is uncertain. Rapid turnaround is essential for personalized vaccine manufacturing, and spatial technologies are generally slower and more expensive than bulk sequencing. Practical implementation will require streamlined workflows and upfront investment.

Third, the prognostic and predictive value of spatial features remains to be validated in prospective trials. We do not yet know whether an antigen that meets all spatial criteria is more likely to yield clinical benefit than one that does not.

Recent methodological advances offer promise. Integration of spatial transcriptomics with single-nucleus RNA sequencing and ATAC-seq has characterized the molecular phenotype of high-grade glioma near the resection margin (38). Separately, high-plex spatial transcriptomics using the CosMx Spatial Molecular Imager has resolved cellular states and interactions in recurrent perivascular niches (23). These studies illustrate complementary routes by which spatial profiling is moving closer to clinically relevant sampling.

Recent integrative datasets, summarized in **Supplementary Table S1**, can also serve as practical reference resources for study design. Atlas-level spatial maps, matrisome profiling, recurrent perivascular niche analysis, PD-1-treated recurrent GBM spatial transcriptomics, and resection-margin multi-omics provide complementary benchmarks for region selection, marker-panel design, cell-state annotation, and immune-accessibility assessment (13, 15, 23, 24, 38). Their value for vaccine development is not that they immediately define validated antigens, but that they provide region-aware frameworks for choosing which tumor areas and cell states should be checked before antigen prioritization.

## Conclusion

Single-cell and spatial omics technologies have transformed our understanding of GBM, revealing a tumor that is spatially organized into distinct malignant cell states and immune microenvironments. Spatial transcriptomics has shown that GBM consists of structured regions with a multi-layered organization associated with hypoxia (10). Spatial proteomics and imaging mass cytometry have mapped the immune landscape at single-cell resolution, identifying cellular neighborhoods associated with survival (18). The integration of these approaches provides a comprehensive picture of GBM as a spatially organized ecosystem.

These findings have direct implications for therapeutic development. The Keskin and GAPVAC trials showed that personalized vaccines can induce T cell responses in GBM, but clinical efficacy has been limited (28, 29). Spatial omics may help address, and more importantly systematically interrogate, three specific gaps: spatial antigen conservation, T-cell accessibility, and microenvironmental suppression. If prospectively validated, integrating spatial information into antigen selection, patient stratification, and response monitoring could move the field from bulk antigen nomination toward spatially informed therapeutic design. The proposed framework and its supporting spatial observations are summarized in **Figure 1** and **Table 1**.

**Figure 1:**
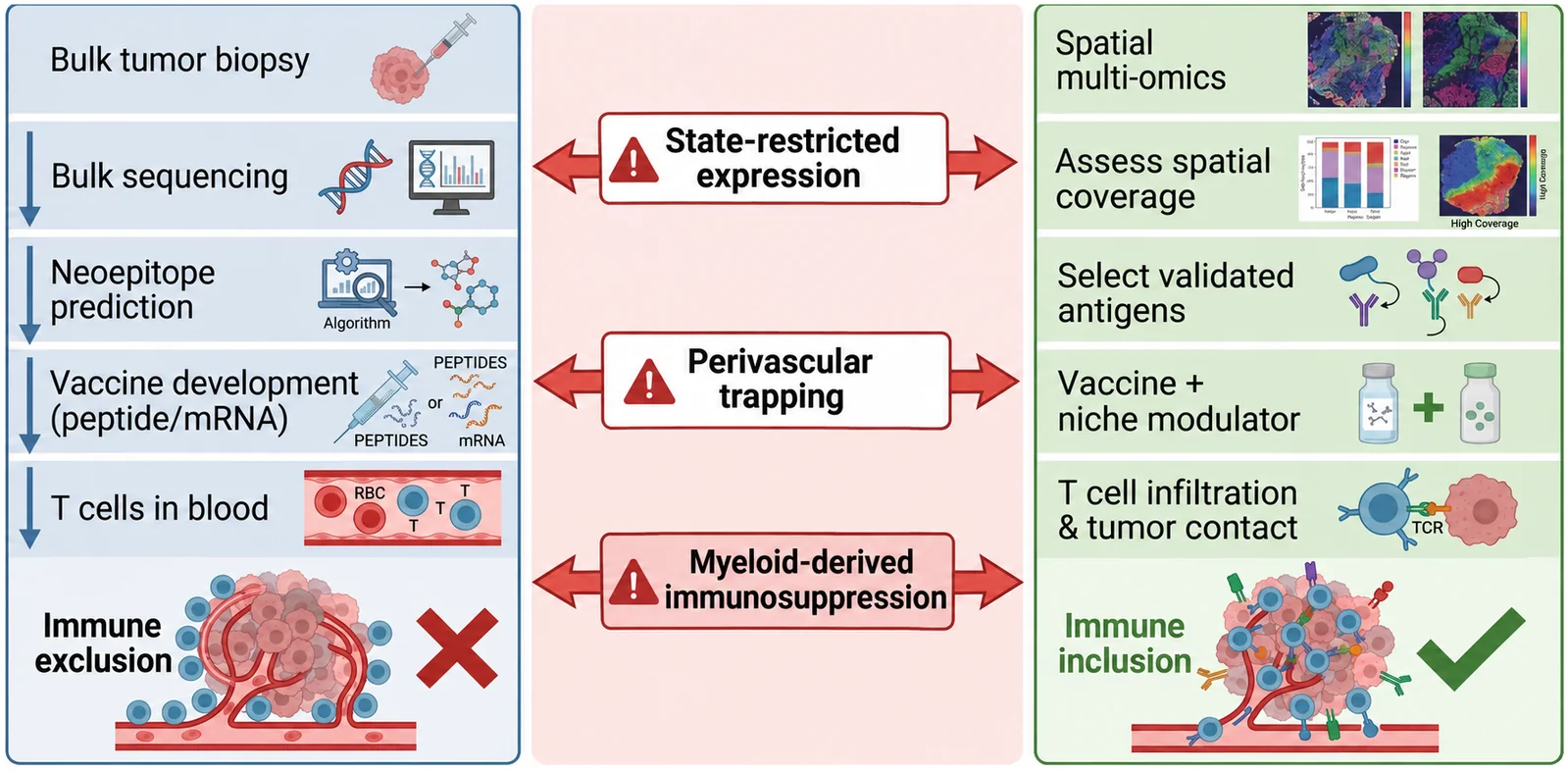
Proposed spatial multi-omic strategy to improve personalized cancer vaccine design in the context of immune exclusion. The left panel illustrates the standard workflow from bulk sequencing to vaccine development, culminating in immune exclusion where T cells are trapped perivascularly. The middle panel highlights three critical biological resistance axes: state-restricted expression, perivascular trapping and myeloid-derived immunosuppression. The right panel illustrates a proposed strategy combining spatial multi-omics, vaccination, and local niche modulators as a testable strategy to promote immune inclusion and deeper tumor infiltration.

**Table 1:** Spatial multi-omic observations and their implications for glioblastoma vaccine immunotherapy.

Spatial omics observation	Biological constraint on vaccine immunotherapy	Spatially informed clinical strategy
Regional segregation of malignant cell states (OPC-like, NPC-like, AC-like, MES-like) across hypoxic, perinecrotic, and perivascular niches (10, 12, 14)	Bulk-derived antigens may be expressed in only a subset of spatially distinct states. Vaccination targeting a single state leaves phenotypically distinct reservoirs untouched, enabling recurrence from state-switched or non-targeted populations.	Multi-state antigen validation. Prioritize antigens validated across spatially distinct regions and malignant states using spatial transcriptomics. Design multivalent vaccines that combinatorially cover spatially separated state repertoires.
Spatial restriction of candidate antigen expression across tumor regions and invasive-margin compartments (14, 16, 23, 38)	Bulk-based antigen nomination can underestimate regional antigen heterogeneity and may select targets present in only a fraction of the tumor.	Multi-region spatial sampling. Incorporate spatial transcriptomics or multisector sampling into pre-vaccine antigen discovery pipelines to help ensure selected antigens are regionally conserved.
Hypoxia-associated cellular organization (10)	An antigen may be transcriptionally expressed in a hypoxic niche but functionally less visible to CD8+ T cells because of local immune suppression or altered antigen-presentation context.	Functional antigen prioritization. Combine spatial transcriptomics with spatial proteomics, multiplexed immunohistochemistry, or immunopeptidomics where possible to test whether antigen expression aligns with protein expression, antigen-presentation machinery, and, where feasible, HLA-bound peptide evidence.
Perivascular T-cell sequestration and parenchymal exclusion (23)	Vaccine-induced T cells may extravasate from the bloodstream but remain trapped in perivascular spaces, unable to penetrate tumor parenchyma where antigen-positive cells reside.	Accessibility-directed combination therapy. Stratify patients by spatial T-cell distribution and test combinations that enhance trafficking or reduce local barriers to parenchymal infiltration.
Spatial co-localization of purinergic signaling components, including tumor CD73 and microglial CD39 (21)	Vaccine-induced T cells reaching antigen-positive cells may encounter an adenosine-rich microenvironment that suppresses proliferation, cytokine production, and cytotoxicity.	Metabolic niche modulation. Spatial mapping of CD73/CD39 proximity can support patient selection for candidate adenosine-pathway combinations, requiring prospective testing.
Therapy-driven phenotypic state switching and recurrent niche reorganization (8, 23)	Vaccine-induced immune pressure may not select only for classic mutational escape; malignant cells may also shift phenotypic states, changing antigen expression without a new DNA alteration.	Dynamic spatial monitoring and adaptive vaccination. Profile recurrent tumors spatially to detect state switching and antigen loss, and prioritize antigens retained across multiple states.

AC, astrocyte-like; MHC, major histocompatibility complex; NPC, neural progenitor-like; OPC, oligodendrocyte progenitor-like; TIL, tumor-infiltrating lymphocyte; MES, mesenchymal-like.

The relevant technologies are increasingly available, although their clinical utility in vaccine trials remains to be established. Spatial transcriptomics can map where antigens are expressed. Spatial proteomics can help confirm whether proteins are present. Imaging mass cytometry can help determine whether T cells can reach antigen-expressing regions and whether the local microenvironment will support their function. The challenge is to incorporate these tools systematically into the next generation of GBM therapeutic trials.
