# Supplementary material for: Spatial heterogeneity in glioblastoma: linking single-cell and spatial multi-omics to vaccine immunotherapy
Source: Front Immunol. 2026 Oct 12;17:1921816. doi: 10.3389/fimmu.2026.1921816 (PMC13697303; doi:10.3389/fimmu.2026.1921816)
Supplement: Supplementary file 1 [file DataSheet1.pdf]

Supplementary Table S1. Recent peer-reviewed integrative single-cell/spatial GBM resources, public data access, and relevance to vaccine-oriented spatial analysis

Study and cohort	Platform and biological focus	Public data access	Use for vaccine-oriented spatial analysis
Sonpatki et al. (2026) (13)<br>Multi-cohort human glioma atlas; Xenium subset from four GBM patients	Visium (17 cases), scRNA-seq/CITE-seq (25 patients), and Xenium (four GBM patients); anatomical niches and vascular/stromal programs	SRA BioProject: PRJNA1337938<br>Zenodo: 10.5281/zenodo.17622242 (Xenium); 10.5281/zenodo.17572905 (Visium)<br>UCSC Cell Browser: multiomic-gbm	Region and cell-state annotation; assessment of vascular/stromal context and malignant-immune proximity before antigen Prioritization
De et al. (2026) (15)<br>Glioma and non-cancerous brain specimens	Xenium targeted single-cell spatial profiling (478-gene panel); ECM/matrisome programs and ligand-receptor networks	EMBL-EBI BioStudies: S-BSST2273	Evaluation of ECM-rich barriers, stromal interactions, and regional constraints on immune trafficking and antigen accessibility
Chanoch-Myers et al. (2026) (16)<br>Patient-derived GBM xenograft models	scRNA-seq, Visium spatial transcriptomics, and multiplexed imaging; local, peri-axonal, and perivascular invasion	GEO: GSE281796<br>Code: github.com/rchanoch/GBM_Invasion_script	Margin-aware validation of antigens across invasive routes and malignant-cell states; preclinical rather than direct patient-tissue evidence
Ajaib et al. (2025) (19)<br>Five paired primary/recurrent IDH-wildtype GBMs	34-marker imaging mass cytometry; three spatial ROIs per tumor; longitudinal cellular architecture	Zenodo: 10.5281/zenodo.14679442<br>Code: github.com/GliomaGenomics/GBM_IMC_Analysis	Assessment of treatment-associated spatial reorganization and recurrent stability of candidate targets at the protein level
Artzi et al. (2025) (24)<br>Matched primary/recurrent IDH-wildtype GBMs, including neoadjuvant nivolumab-treated cases	GeoMx Whole Transcriptome Atlas profiling of SOX2-positive tumor and IBA1-positive myeloid compartments	Zenodo: 10.5281/zenodo.16839828	Spatial pharmacodynamic benchmarking and evaluation of whether immunotherapy remodels tumor and myeloid compartments
Pai et al. (2025) (38)<br>Four paired core/margin HGGs for single-nucleus multiome plus two additional HGGs for spatial transcriptomics	Paired core/margin snRNA-seq and snATAC-seq, integrated with Visium spatial transcriptomics from two additional specimens	GEO: GSE286413 (spatial transcriptomics); GSE286415 (snATAC-seq); GSE286419 (snRNA-seq)	Region selection and validation of antigen conservation across tumor core and infiltrative margin

Abbreviations: CITE-seq, cellular indexing of transcriptomes and epitopes by sequencing; ECM, extracellular matrix; GBM, glioblastoma; HGG, high-grade glioma; IDH, isocitrate dehydrogenase; ROI, region of interest; scRNA-seq, single-cell RNA sequencing; snATAC-seq, single-nucleus assay for transposase-accessible chromatin sequencing; snRNA-seq, single-nucleus RNA sequencing.
